# Novel approaches for neuroprotection: Focus on neurodegenerative and ischemic central nervous system diseases

**DOI:** 10.1002/nep3.70045

**Published:** 2026-06-18

**Authors:** Shen Li, Xunming Ji, Piotr Walczak, Johannes Boltze

**Affiliations:** ^1^ Department of Neurology and Psychiatry, Beijing Shijitan Hospital Capital Medical University Beijing China; ^2^ Beijing Institute of Brain Disorders Capital Medical University Beijing China; ^3^ Chinese Academy of Medical Sciences and Peking Union Medical College Beijing China; ^4^ Department of Diagnostic Radiology and Nuclear Medicine, Center for Advanced Imaging Research University of Maryland Baltimore Maryland USA; ^5^ School of Life Sciences University of Warwick Coventry UK

**Keywords:** Alzheimer's disease, cerebral ischemia, ischemic stroke, mechanotransduction, muscle–brain crosstalk, neurodegeneration, neuroinflammation, Parkinson's disease, ursodeoxycholic acid

There is still a lack of causative treatments for many central nervous system (CNS) diseases despite increasing research efforts. This specifically applies to neurodegenerative and cerebrovascular conditions. Neuro‐ and cerebroprotective approaches receive great attention in this field as they offer the potential to at least mitigate acute disease impact or slow down disease progression in chronic conditions. Neuro‐ and cerebroprotective approaches can be applied with great flexibility in therapeutic settings, as they can represent a primary treatment approach but also an adjuvant, secondary treatment with the aim to increase the impact of a primary intervention. A prominent example is ischemic stroke, in which neuroprotective paradigms now are used to support recanalization.^1^ The present issue of *Neuroprotection* focuses on research investigating neuroprotective approaches in two of the most frequent neurodegenerative conditions, Alzheimer's disease (AD) and Parkinson's disease (PD). It also features research on ischemic stroke. The presented work spans from experimental mechanistic investigations over reviews to the suggestion of new pathomechanistic concepts that may offer opportunities to access new therapeutic targets.

Microglia as the resident immune cells of the brain are known to play important regulatory and effector roles in neuroinflammation, neurodegeneration, and neuroprotection. Lei and colleagues suggest a new concept describing the role of microglia in very early stages of AD. According to the established amyloid hypothesis, an imbalance between the production and removal of amyloid β42 (Aβ42), causing an accumulation of Aβ42, may initiate AD. The accumulation can be caused by an increased production and/or a reduced clearance of Aβ42. The concept presented by Lei et al. expands this classic hypothesis. Based on experimental data, they describe a compensatory network of microglia, astroglia, and oligodendroglia, in which microglia play a central role. This compensatory network mediates resilience against Aβ pathology by preserving protein homeostasis and neuronal health. While the network would initially counterbalance the impact of Aβ, a loss of its compensatory function is supposed to lead to irreversible pathology, which is further amplified by positive feedback loops. This will lead to the onset of clinical AD characterized by its typical cognitive symptoms. If the concept is verified by further experimental studies, it will have important implications as it would provide novel targets for AD prevention and therapy. The authors suggest applying dynamical systems approaches for further investigation of the compensatory network, and to detect potential therapeutic mechanisms and related time windows for intervention. This is well in line with previously published initiatives for improving prevention, diagnosis, and treatment of AD.^2^

PD is a chronic and progressive neurodegenerative disorder without a curative treatment and is characterized by typical motor symptoms but also a wide spectrum of non‐motor symptoms. Páez‐García and colleagues present a narrative review focusing on the non‐motor symptoms and highlighting the importance of muscular health in PD. It has been known that sarcopenia and impaired muscle health are prevalent among PD patients and represent both a risk factor and a cause for frailty and falls in PD. Similar to physiological sarcopenia during individual aging, clinical data show that regular aerobic exercise improves balance and gait in PD patients but also neuropsychological aspects such as mood and cognition. This is important because depression and cognitive decline are impactful symptoms especially in late‐stage PD. The authors do not only review clinical data but also provide mechanistic insights based on the literature. Exercise releases signaling molecules known as exerkines. Interestingly, these comprise not only factors related to muscular growth and muscle bulk maintenance such as insulin‐like growth factor 1 or myostatin, but also brain‐derived neurotrophic factor (BDNF). This suggests a muscle–brain crosstalk in the steady state but also in PD, which can be augmented and improved by exercise. Moreover, many exerkines contribute to anti‐inflammatory, antioxidant, and metabolic regulatory pathways, which can help to promote neuronal resilience and preserve synaptic plasticity in PD but also in other neurodegenerative conditions. This knowledge can inform individualized training programs as an adjuvant, non‐pharmacological intervention in PD treatment.

López‐Pérez and coworkers present original research describing an interesting therapeutic approach for PD using lyophilized bee venom. Bee venom has been used to treat inflammatory conditions and is well characterized for its active components including apamin and phospholipase A2. The authors used the well‐established PD model of 6‐hydroxydopamine (6‐OHDA) injections into the dorsomedial striatum in mice. Animals were followed up for 30 days. Treatment paradigms included treatment with l‐DOPA and carbidopa as well as l‐DOPA/carbidopa plus bee venom from Day 13 after lesion induction. Treatment groups were compared against untreated controls and sham animals. The combination treatment improved motor symmetry in forelimbs and mitigated contralateral paw dragging. Moreover, it improved cognitive performance in the novel object recognition test, which assesses episodic memory. It also improved performance in the corridor test, a complex measure of motor function reflecting striatal dopaminergic depletion. Although l‐DOPA/carbidopa treatment already improved functional outcome in PD mice, the combination treatment with bee venom induced even stronger effects that were statistically significant in most test conditions. Further research is required on the exact mechanisms by which this therapeutic effect is induced, in particular at the cellular and molecular levels. Moreover, the identification of the active compound(s) in bee venom mediating the therapeutic benefit may ease further translational and potential clinical research on this new PD treatment paradigm.

Ursodeoxycholic acid (UDCA) levels are reduced in several neurodegenerative disorders and stroke. Moreover, UDCA can easily cross the blood–brain barrier (BBB). It has even been used in a randomized and placebo‐controlled Phase II trial in PD revealing the safety and tolerability of the approach.^3^ The review by Chong et al. focuses on a potential role of UDCA as an experimental treatment in PD and AD. The review starts with a short overview of PD and AD highlighting shared pathomechanisms including protein aggregation, neuroinflammation, impaired auto‐ and mitophagy, mitochondrial dysfunction, as well as oxidative stress. Second, it features a detailed description of UDCA explaining its structure, pharmacokinetics, and safety profile. Although higher UDCA concentrations can cause abdominal discomfort, there are no side effects affecting the CNS. The main part of the review focuses on literature reporting the experimental use of UDCA in PD and AD as well as on clinical trials of UDCA in PD or its potential for clinical use in AD, respectively. This comprises a detailed description of potential modes of action revealed in cell culture and animal experiments, indicating that UDCA can mitigate shared pathomechanisms of PD and AD as described above. The review also transparently discusses the limitations of previous and current approaches as well as existing knowledge gaps. It finally points at potential future research directions such as the use of UDCA in combination therapies, in personalized medicine approaches, or the discovery of options for improved delivery.

Ischemic stroke is a prevalent and devastating condition that has been a dominant topic in previous issues of *Neuroprotection*.^4^ A focused experimental study by Gallagher et al. provides further mechanistic insights into stroke pathophysiology and a potential new therapeutic target. The work focused on aromatase, a key enzyme in the biosynthesis of 17β‐estradiol, a highly potent estrogen with neuroprotective effects. The time course of aromatase expression was investigated in ovariectomized female rats showing increased expression in the infarct boundary zone and the hippocampus 24 h after permanent middle cerebral artery occlusion (MCAO) in some, but not all readout parameters used. Male mice overexpressing aromatase had increased 17β‐estradiol plasma levels but did neither exhibit better stroke outcome nor reduced lesion sizes. Subcutaneous injecting 10 μg of the aromatase inhibitor letrozole resulted in decreased 17β‐estradiol plasma levels but did not aggravate stroke outcome. These findings indicate a potential role of aromatase after stroke, which requires more mechanistic investigation as manipulating aromatase expression neither resulted in beneficial nor detrimental effects after MCAO. A valuable feature of the study is the use of hypertensive animals, increasing the external validity of the study. Hypertension is a frequent and relevant comorbidity of stroke and not only increases the risk of suffering from stroke but can also aggravate stroke‐related secondary process causing further brain damage. A prominent example is the post‐stroke inflammatory response.^5^

Neuro‐ and cerebroprotective approaches are relevant adjuvant treatments to recanalization approaches, which are believed to have the potential to augment the impact of recanalization.^6^ Such combination approaches are therefore the subject of many translational and clinical studies.^7^ The study by Schneider and colleagues investigated the suitability of the mammalian target of rapamycin complex 1 (mTORC1) inhibitor rapamycin for such approaches. Rapamycin reportedly has neuroprotective effects but was predominantly administered prior to or during stroke induction in previous experimental studies. Such protocols aid mechanistic investigations but are of limited translational value. The authors used 90 min of transient MCAO in rats, which is a well‐established method of mimicking ischemic stroke with subsequent recanalization by thrombectomy.^8^ Rapamycin significantly improved functional outcome and decreased lesion volumes. However, there were no effects on edema and BBB integrity, and rapamycin did not affect cerebral blood flow (CBF) after recanalization. Rapamycin reduced the ratio between phosphorylated and total mTOR, but the difference was not statistically significant 3 days after MCAO. These findings require further investigation since several aspects could explain these observations. For instance, there may be species differences in susceptibility to rapamycin dose, the unintended use of a subcritical dose, or rapamycin effects at earlier time points.

Mechanosensitive ion channels have long been an “underinvestigated” area of neuroscience. One of the most prominent mechanosensitive ion channels is Piezo‐1. Piezo is expressed throughout the body and contributes to regulating CBF, neuronal signal conduction, and synaptic plasticity in the CNS. It is also considered to be important for the response to endothelial shear stress. Liu et al. present a bibliometric analysis of research on Piezo‐1 including 173 studies published throughout a period of almost 12 years. While the overall quantity is still limited, the analysis revealed that there is currently a steep increase in annual research output on Piezo‐1. Moreover, publications are appearing in high‐impact journals, which can be considered an indicator of good research quality. Areas of interest were mechanotransduction and neuroinflammation. However, not many studies investigate potential translational aspects. The review addresses this aspect by suggesting future research directions and aims of translational research.

This issue of *Neuroprotection* also features the first Letter to the Editor. In their letter, Kokiwar et al. provide meaningful comments on a previously published article describing a potential role of vitamin E for the prevention of intraventricular hemorrhage in preterm neonates.^9^ The letter acknowledges the potential value of the approach but also points out limitations in practical use and existing knowledge gaps. These comments contribute meaningfully to the critical discussion of the topic and clearly help to depict a more holistic picture. We were delighted to receive this letter not only because of its scientific relevance but also because it shows that research published in *Neuroprotection* is increasingly discussed in the scientific community.

The present issue of *Neuroprotection* again provides a collection of original and review articles with a strong focus on neuroprotection in AD, PD, and stroke. The presented studies are well in line with contemporary trends in the scientific community such as investigating neuroprotective approaches in the context of recanalization therapies for stroke or the search for novel therapeutic targets in AD. The focus is on both basic and translational activities, the latter often with clear perspectives towards a potential clinical translation. Thus, the issue should be of interest to many colleagues in experimental and clinical neuroscience. The issue also contains the first Letter to the Editor.

As we release our June issue, we are thrilled to share the news we've all been waiting for: *Neuroprotection* has officially received its debut Impact Factor: 4.1, ranking in Q2 (106/330) in Neuroscience. Since indexing in all major scientific databases has been completed, this will significantly increase the journal's visibility. Indicators related to the impact factor such as the CiteScore showed a continuous and steep increase over the last few years. We are very happy with journal's debut impact factor and think this is respectable for a new journal. Despite these major developments, publication services remain completely free of costs for authors, most likely until the end of the year. With the first impact factor being announced, we also expect a noticeable increase in the number of submissions to our journal, and we do look forward to these future contributions.

## AUTHOR CONTRIBUTIONS

All authors listed have conceptualized, written and edited the publication, and approved its final version for submission.

## CONFLICT OF INTEREST STATEMENT

X.J., P.W., and J.B. are Co‐Editors in Chief of *Neuroprotection*. S.L. serves as an executive editor for the journal. All authors were blinded from reviewing or making decisions on the manuscript. The article was subject to the journal's standard procedures, with peer review handled independently of the Co‐Editors in Chief and their research groups. P.W. is a founder and holds equity in IntraArt, LLC and Ti‐com, LLC.
